# Cysteine-Rich Secretory Protein-3 (*CRISP3*) Is Strongly Up-Regulated in Prostate Carcinomas with the *TMPRSS2-ERG* Fusion Gene

**DOI:** 10.1371/journal.pone.0022317

**Published:** 2011-07-21

**Authors:** Franclim R. Ribeiro, Paula Paulo, Vera L. Costa, João D. Barros-Silva, João Ramalho-Carvalho, Carmen Jerónimo, Rui Henrique, Guro E. Lind, Rolf I. Skotheim, Ragnhild A. Lothe, Manuel R. Teixeira

**Affiliations:** 1 Department of Genetics, Portuguese Oncology Institute-Porto, Porto, Portugal; 2 Department of Pathology, Portuguese Oncology Institute-Porto, Porto, Portugal; 3 Cancer Genetics Group, Research Centre of the Portuguese Oncology Institute-Porto, Porto, Portugal; 4 Cancer Epigenetics Group, Research Centre of the Portuguese Oncology Institute, Porto, Portugal; 5 Department of Pathology and Molecular Immunology, Institute of Biomedical Sciences Abel Salazar (ICBAS), University of Porto, Porto, Portugal; 6 Department of Cancer Prevention, Institute for Cancer Research, Norwegian Radium Hospital, Oslo University Hospital, Oslo, Norway; 7 Centre for Cancer Biomedicine, Faculty of Medicine, University of Oslo, Oslo, Norway; University of Nebraska Medical Center, United States of America

## Abstract

A large percentage of prostate cancers harbor *TMPRSS2-ERG* gene fusions, leading to aberrant overexpression of the transcription factor *ERG*. The target genes deregulated by this rearrangement, however, remain mostly unknown. To address this subject we performed genome-wide mRNA expression analysis on 6 non-malignant prostate samples and 24 prostate carcinomas with (n = 16) and without (n = 8) *TMPRSS2*-*ERG* fusion as determined by FISH. The top-most differentially expressed genes and their associations with *ERG* over-expression were technically validated by quantitative real-time PCR and biologically validated in an independent series of 200 prostate carcinomas. Several genes encoding metabolic enzymes or extracellular/transmembrane proteins involved in cell adhesion, matrix remodeling and signal transduction pathways were found to be co-expressed with *ERG*. Within those significantly over-expressed in fusion-positive carcinomas, *CRISP3* showed more than a 50-fold increase when compared to fusion-negative carcinomas, whose expression levels were in turn similar to that of non-malignant samples. In the independent validation series, *ERG* and *CRISP3* mRNA levels were strongly correlated (r_s_ = 0.65, p<0.001) and both were associated with pT3 disease staging. Furthermore, immunohistochemistry results showed CRISP3 protein overexpression in 63% of the carcinomas and chromatin immunoprecipitation with an anti-ERG antibody showed that *CRISP3* is a direct target of the transcription factor ERG. We conclude that *ERG* rearrangement is associated with significant expression alterations in genes involved in critical cellular pathways that define a subset of locally advanced PCa. In particular, we show that *CRISP3* is a direct target of ERG that is strongly overexpressed in PCa with the *TMPRSS2-ERG* fusion gene.

## Introduction

Gene fusions involving members of the ETS family of transcription factors, such as *ERG*, *ETV1*, *ETV4* and *ETV5*, have been shown to occur in a high proportion of prostate carcinomas [1], [2], [3]. *ERG* rearrangement with *TMPRSS2*, a prostate-specific transmembrane serine protease regulated by androgens [4], accounts for more than 90% of the fusion-positive cases, being present in about 50% of prostate carcinomas [5] and 20% of the presumed precursor lesion high-grade prostatic intraepithelial neoplasia (HGPIN) [6]. ETS rearrangements seem to define a distinct subgroup of prostate carcinomas, but their biological relevance and clinical impact is not yet understood.

ETS transcription factors have been associated with several biological processes [7], [8], [9]. Identification of deregulated genes downstream of the overexpressed *TMPRSS2*-*ERG* fusion gene may clarify the relevance of this event for prostate carcinogenesis and provide feasible targets for novel treatment approaches. The scarce studies that have addressed this issue have described only a limited number of genes associated with *ERG* overexpression in prostate cancer [10], [11], [12]. Using an *in silico* approach on published expression data, it has been shown that *HDAC1* (a histone deacetylase involved in epigenetic programming) was consistently co-expressed with *ERG* [10]. These authors also highlighted genetic signatures enriched in *ERG* positive tumors, namely an increased expression of WNT-associated pathways and down-regulation of TNF and cell-death pathways [10]. Increased expression of members of plasminogen activator pathway were also described to be associated with ERG overexpression [11]. Using a similar approach to provide signatures linked to ETS transcription factors (*ERG*, *ETV1* and *ETV4*), others have reported an enrichment of genes of the chromosome region 6q21 when comparing ETS-negative with ETS-positive PCa [12].

In this work, the transcriptomes of a series of prostate carcinomas, stratified by the *TMPRSS2-ERG* fusion gene status, were analyzed using whole-genome expression microarrays. Genes with significant differential expression between the *TMPRSS2-ERG* positive and negative lesions were identified and validated by qRT-PCR in a larger series of prostate carcinomas, as well as by immunohistochemistry and chromatin immunoprecipitation analyses (ChIP).

## Methods

### Ethics Statement

This study was approved by the institutional review board.

### Prostate tissue specimens

Primary tumor samples were collected from patients with clinically localized prostate adenocarcinoma (PCa) consecutively diagnosed and treated with open radical prostatectomy at the Portuguese Oncology Institute – Porto, Portugal. For control purposes, benign prostate hyperplasias (BPH) and normal prostate tissues (NPT) were used (grouped as non-malignant tissues – NMT). BPH samples were collected from patients that underwent transurethral resection of the prostate and NPT samples were collected from the peripheral zone of prostates obtained from cystoprostatectomy specimens of bladder cancer patients. Two series of primary prostate carcinomas were available for the purposes of this study: a test group of 24 carcinomas diagnosed from 1999 to 2000, and a validation group comprising 200 consecutive carcinomas collected from 2001 to 2004. From each case, a representative paraffin block of the dominant tumor focus was selected for FISH and immunohistochemical analysis. The tumor areas varied from 0.5 to 2.5 cm in greatest diameter, approximately. After histological identification of PCa, BPH and NPT by an experienced pathologist (author: RH), fresh-frozen tissue fragments (which were immediately frozen after surgical removal, i.e., less than 30 minutes following surgery) were trimmed to maximize the yield of target cells (>70%) and an average of fifty 12-micron thick sections was cut for RNA extraction. Relevant clinical data, namely Gleason grading, clinico-pathological staging and PSA level at diagnosis, were obtained from medical records.

### Expression microarrays

A total of 24 PCa, 3 BPH and 3 NPT (collected from the peripheral zone and selected based on availability of good quality RNA) were submitted to whole-genome expression analysis using the Applied Biosystems Expression Array platform (Applied Biosystems, Foster City, CA, USA). For this purpose, total RNA was extracted from 250 mg of tissue using the FastRNA Kit (Qbiogene, Carlsbad, USA) and processed into digoxigenin(DIG)-labeled cDNA using the Applied Biosystems Chemiluminescent RT Labeling Kit according to the manufacturers' instructions. The Human Genome Survey Microarray slides (V2.0) contain 32,878 oligonucleotide probes (60-mers) targeting expressed sequences of more than 29,000 known or predicted genes. The system includes dedicated software for the normalization, processing and statistical analysis of the acquired images. Normalized, log-transformed and median-centered array results (features with a signal at least two standard deviations above the local noise level in at least 50% of the samples) were submitted to Significance Analysis of Microarrays (SAM) using the two-class unpaired t-statistic method to determine differentially expressed genes among sample subgroups [13].

### Fluorescence in situ hybridization (FISH)

To determine *TMPRSS2-ERG* fusion status in the test series of carcinomas (n = 24) and in the non-malignant tissues (n = 6), four-micron thick sections were cut from representative paraffin-embedded blocks of each sample onto SuperFrost Plus Adhesion slides (Menzel-Glaser, Braunschweig, Germany). Sample processing, hybridization, and analysis were performed as previously described [14]. A triple-labeled commercial probe flanking the *TMPRSS2* and *ERG* genes at 21q22 (Poseidon *TMPRSS2-ERG* probe, Kreatech Diagnostics, Netherlands) was applied to each sample. The probe design allows identification of *TMPRSS2-ERG* fusions but also possible rearrangements of each gene with other partners. An abnormal signal pattern was considered representative when present in a minimum of 50 morphologically intact, nonoverlapping nuclei [15].

### Technical validation by quantitative Real-time PCR (qRT-PCR)

To confirm findings obtained in the expression array, qRT-PCR was performed for selected genes in a subset of 13 samples with available RNA (3 NMT and 10 PCa from the series analyzed with the array). For this purpose, 200 ng of RNA were converted into cDNA using the TransPlex Whole Transcriptome Amplification Kit (Sigma-Aldrich), according to the manufacturer's instructions. Primers and probes for *ERG*, *CRISP3* and *RBMS2* were designed using the Primer Express 2.0 software (Applied Biosystems) and acquired from Metabion (Metabion, Martinsried, Deutschland) (Table S1). Primers and probe for the beta-glucuronidase (*GUSB*) gene, used as endogenous control, were acquired as a pre-developed assay reagent from Applied Biosystems. To determine the relative expression level of each target gene, the comparative Ct method was used [16].

### Biological validation using Taqman Low Density Arrays (TLDA)

To validate selected candidate genes, an independent series of 200 consecutive PCa cases was analyzed using custom-design TLDA cards from Applied Biosystems, specifically comprising probes for *ERG*, *CRISP3* and *RBMS2*. For this purpose, 100 µg of RNA were converted to cDNA using the High-Capacity RNA-to-cDNA kit, according to the manufacturer's instructions (Applied Biosystems). Relative expression values were obtained by the comparative Ct method, using *18S* as endogenous control.

### External validation from publicly available microarray data

Using the data available from Setlur *et al.* (dataset GSE8402 [17]) that stratifies 455 primary prostate tumours according to the presence/absence of the *TMPRSS2-ERG* fusion, we selected all the 103 fusion positive cases available and also 103 randomly selected fusion negative cases. Normalized signal intensity values for both *ERG* and *CRISP3* were linearized and plotted in both *TMPRSS2-ERG* positive and negative groups.

### Chromatin immunoprecipitation (ChIP)

We used the *TMPRSS2-ERG* positive cell line VCaP (European Collection of Cell Cultures, Sigma-Aldrich) and the ERG monoclonal antibody #EPR3864 (Abcam) to evaluate ERG binding to *CRISP3* promoter. For each immunoprecipitation with the EZ-Magna ChIP™ G kit (Millipore) 2×10^6^ cells were used following the manufacturer's instructions. To select for putative ETS binding sequences in the promoter region of *CRISP3*, a bioinformatic survey of the 10 kb sequence upstream of the *CRISP3* ATG start site was conducted using ConSite [18]. Three regions, each containing two putative ETS binding sequences, were selected for PCR analysis of the ERG-immunoprecipitated chromatin. Primers were designed using the Primer3 online software, and acquired from Metabion (Metabion, Martinsried, Deutschland) (Table S2). The presence of the *TMPRSS2-ERG* rearrangement in VCaP cells was confirmed by FISH analysis using the triple-labeled Poseidon *TMPRSS2-ERG* probe, as described above. High levels of the *TMPRSS2-ERG* transcript were confirmed by qRT-PCR (data not shown).

### Protein analysis by immunohistochemistry (IHC)

Four-micron thick sections from representative paraffin-embedded blocks of the 30 samples used in the expression array (24 PCa and 6 NMT) were deparaffinised in xylene and hydrated through an alcohol series. After antigen retrieval with EDTA, tissues were stained with anti-CRISP3 antibody (clone LV-2A2, sc-101378) diluted 1/200, as previously described [19]. Antibody specificity was confirmed by Western-blot analysis. An additional 10 BPH and 8 NPT samples were included to increase the number of negative controls, whereas pancreatic tissue was used as a positive control (data not shown). Protein expression was classified according to the following parameters: 0- no immunoexpression, 1- underexpression, 2- expression similar to the normal tissue, 3- overexpression. Cases with heterogeneous expression were also noted.

### Statistical analysis

The non-parametric Mann-Whitney (MW) test was applied to compare RNA expression levels of *ERG*, *CRISP3* and *RBMS2* in the different sample groups [non-malignant tissue (NMT), *TMPRSS2-ERG*-positive PCa (TMP-ERG^+^) and *TMPRSS2-ERG*-negative PCa (TMP-ERG^−^)] and to correlate this expression with clinico-pathological parameters. To assess possible associations between *ERG*, *CRISP3* and *RBMS2* levels in the same samples, and to determine the concordance of findings obtained by different methodologies, the Spearman non-parametric correlation test (r_s_) was used. For correlation analysis between the IHC data and the clinico-pathological parameters, the Pearson Chi-Square was used, testing for Linear-by-linear association when appropriate. A *p-*value smaller than 0.05 was considered statistically significant. Statistical analyses were performed using the Statistical Package for Social Sciences software, version 15.0 (SPSS Inc., Chicago, IL).

## Results

### Fluorescence in situ hybridization

**Figure 1:**
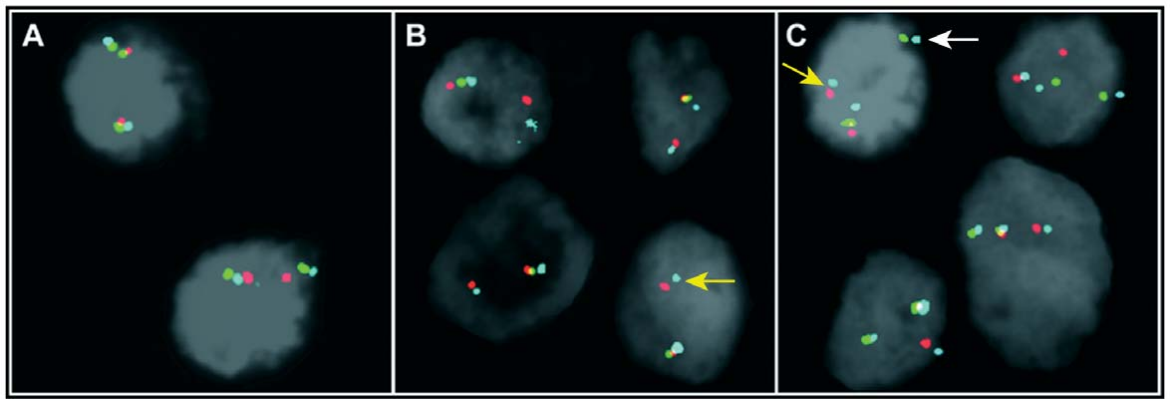
Examples of the *TMPRSS2-ERG* tri-color FISH assay results. A) Two clusters of three co-localized signals indicative of a sample without rearrangement (# P134); B) One cluster of three co-localized signals and one cluster with only the blue and red signals, indicative of rearrangement through deletion (# P164); C) One cluster of three co-localized signals and two clusters (blue-green and blue-red) indicative of rearrangement through insertion (# P072). Yellow arrowheads point to the *TMPRSS2-ERG* fusion; insertion of the segment between *TMPRSS2* and *ERG* in a different part of the genome is marked by a white arrowhead. Split of the blue signal was consistently found in *TMPRSS2-ERG* rearrangement through insertion, as the blue probe covers part of *ERG* (also validated with *ERG* break-apart BAC probes in the same cases – not shown). Detailed FISH findings are available in Table S3.

Sixteen of the 24 carcinomas analyzed had FISH signal patterns indicative of a *TMPRSS2-ERG* rearrangement (67%, Table S3, Figure 1). Based on the three-color probe setting, 8 PCa showed a normal signal pattern (Figure 1A), 5 PCa displayed a pattern consistent with interstitial deletion between the *TMPRSS2* and *ERG* genes (Figure 1B), whereas 11 PCa showed the insertion mechanism of the rearrangement (Figure 1C). None of the samples showed a pattern indicative of *ERG* or *TMPRSS2* involvement with other partners. No rearrangement was seen in the six non-malignant samples.

### Expression microarray analysis

After quantile normalization of the expression results for the 30 samples, a total of 18,797 probes passed our final quality criteria (signal intensity more than two standard deviations above the local noise level in at least 12 samples) [20]. It should be noted that the values for the *ERG* probe in the expression array showed a modest variation between fusion-positive and fusion-negative cancers. This particular 60-mer probe targets an exon11:exon12 junction towards the 3′ terminal of *ERG* that is common to most transcripts. As the targeted sequence shows no known single base polymorphisms, the probe should be able to detect fusion-driven overexpression, even if this was not evident in our data. Given that qRT-PCR with a different probe design clearly validated *ERG* overexpression in fusion-positive carcinomas (see corresponding Results section below), fusion status as determined by FISH was used for subsequent SAM analysis.

**Figure 2:**
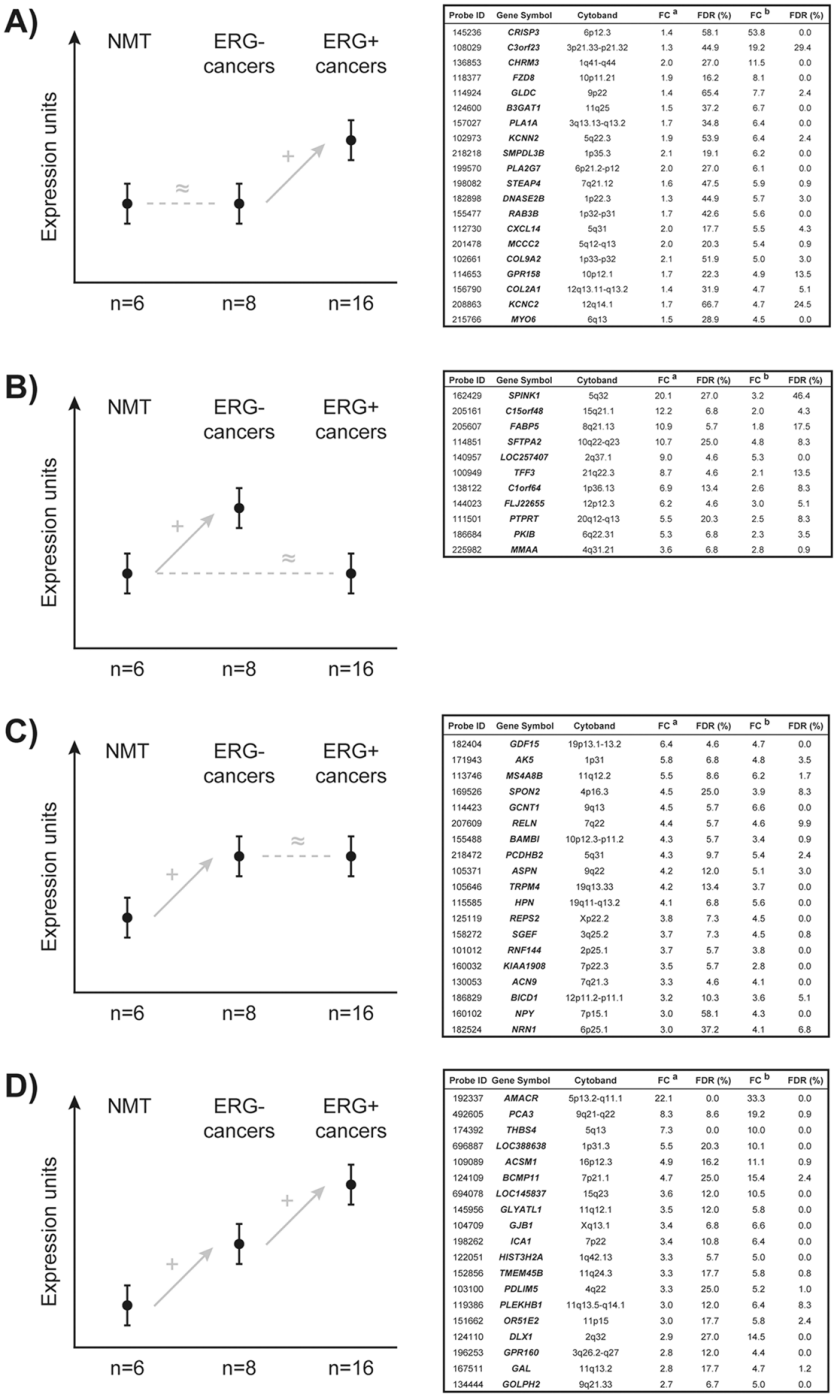
Genes showing different patterns of overexpression in carcinomas. A) genes with high fold-increase in *ERG*-positive carcinomas; B) genes with overexpression in *ERG*-negative carcinomas; C) genes with high fold-increase in carcinomas, independent of *ERG* status; D) genes with high fold-increase in ERG-negative carcinomas accompanied by an even greater overexpression in ERG-positive cancers. Abbreviations: FC(a), median fold-change between non-malignant samples (NMT) and ERG-negative carcinomas; FC(b), median fold-change between non-malignant samples and ERG-positive carcinomas; FDR, false discovery rate. The top 20 genes in each subgroup, ranked based on fold-increase, are provided (when available).

Several gene lists were generated from the normalized, log-transformed data using SAM (two-class unpaired analysis, t-statistic). On a first analysis, cancerous (n = 24) and non-cancerous lesions (n = 6) were compared, providing ∼1,596 significant hits at a 5% false-discovery rate (FDR). Genes with significant differences between *ERG*-positive (n = 16) and *ERG*-negative tumors (n = 8) were also obtained (114 hits, FDR = 5.3%). A comparison of non-malignant samples with either *ERG*-positive (1154 hits, FDR = 5%) or *ERG*-negative cancers (35 hits, FDR<5%) was additionally performed. By cross-tabulating the aforementioned gene lists, several candidates emerged that were categorized into subgroups based on their distinct biological roles (Figure 2 and Figure S1).

The first subgroup of genes showed significant fold-changes only in the presence of over-expressed *ERG* (Figure 2A and Figure S1A). Strikingly, the top-ranked gene – *CRISP3* – showed a massive fold increase in *ERG*-positive carcinomas as compared to both non-malignant tissue and *ERG*-negative carcinomas, which led us to validate and study this candidate target further. Within this list, comprised mainly of over-expressed candidates, several metabolic enzymes could be found, such as *GLDC* (amino-acid metabolism), *B3GAT1* (carbohydrate metabolism), *PLA1A* (lipid, fatty acid and steroid metabolism), *PLA2G7* (fatty acid and steroid metabolism) and *DNASE2B* (nucleic acid metabolism). Several membrane receptor and extracellular matrix proteins were also noticeable, with strong up-regulation of *COL9A2* (cell adhesion and communication functions), *COL2A1* (cell adhesion and motility), *KCNC2* (ion channel, cell communication), *KCNN2* (ion channel, cell communication), *MYO6* (cell structure and motility), *CHRM3* (membrane receptor with signaling activity) and *RAB3B* (intracellular protein trafficking, signaling transduction function) (Figure 2A). The top-ranked down-regulated genes, such as *HSPB3* (chaperone) or *HIF3A* (transcription factor), displayed much smaller fold-changes (Figure S1A).

The second subgroup comprised genes that showed a mutually exclusive association with *ERG* (Figure 2B and Figure S1B). Within this group, *SPINK1* (serine protease inhibitor), *TFF3* (growth factor, signaling molecule), *PKIB* (protein kinase inhibitor) and *FABP5* (carrier protein, steroid hormone-mediated signaling) showed noticeably higher levels of expression in samples without *ERG* fusion (Figure 2B), whereas *NUCB1* (nucleic acid binding protein), *ORM1* (binding protein) and *GRN* (signaling molecule) showed significantly lower expression in this *ERG*-negative group (Figure S1B).

We then distinguished a group of genes with a significant fold-increase in carcinomas and whose expression changes did not seem to be associated with *ERG* (Figure 2C and Figure S1C). Noteworthy hits based on fold-change and function were *AK5* (a kinase involved in nucleic acid metabolism), *RELN* (serine protease), *ASPN* (transmembrane receptor with signal transduction activity), *HPN* (serine protease) and *REPS2* (protein modulator, part of signal transduction complex) (Figure 2C). Within the list of genes significantly down-regulated in tumor samples (but not associated with *ERG*), *CXCL13* (cytokine precursor), *UBOX5* and *ZNF179* (both showing ubiquitin ligase activity) are worth highlighting (Figure S1C).

Finally, a subgroup of genes showed significant fold-differences in *ERG*-negative carcinomas with an even more significant increase/decrease in *ERG* positive tumors (Figure 2D and Figure S1D). Within the very few genes showing under-expression in *ERG*-negative carcinomas with an even more marked fold-decrease in *ERG*-positive lesions, *RBMS2* (nucleic acid binding protein) displayed a massive fold-change reduction, which we set out to validate (Figure S1D). Within the group of genes showing the inverse pattern (*i.e*., overexpression in *ERG*-negative cancers with a marked fold-increase in *ERG*-positive tumors), noteworthy hits were *AMACR* (lipid and amino-acid metabolic enzyme), *PCA3* (prostate cancer antigen), *THBS4* (a membrane protein involved in various processes) and *GAL* (signaling molecule) (Figure 2D).

### qRT-PCR analysis (technical validation)

**Figure 3:**
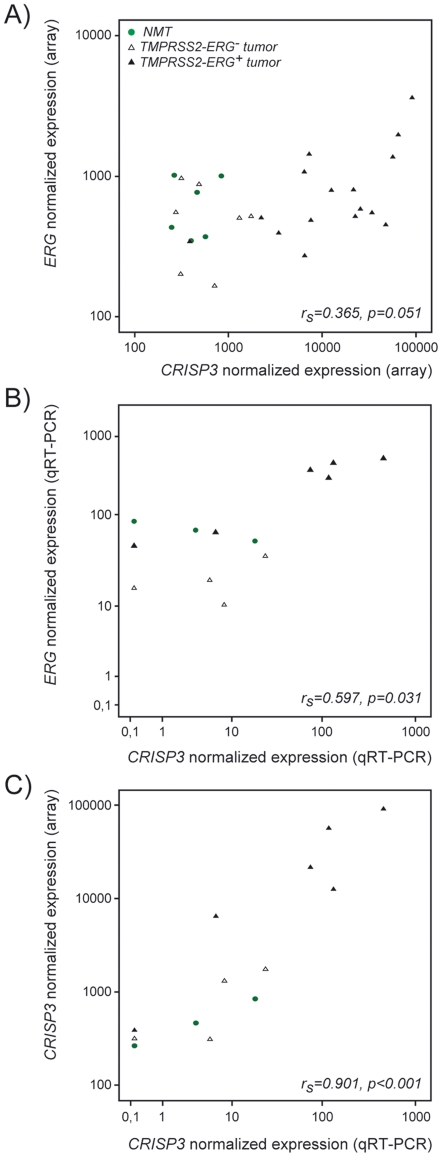
Correlation between *ERG* and *CRISP3* in the test series of samples. A) Expression array findings for *ERG* and *CRISP3*; B) Quantitative Real-time PCR findings for *ERG* and *CRISP3*; C) Methodological comparison between array and qRT-PCR probes for *CRISP3*.

The main findings obtained in the technical validation series are displayed in Figure 3 and Figure S2, together with the array results for *ERG* and *CRISP3*. Within this 13 sample subset, a positive correlation could be seen between *CRISP3* and *ERG* values (r_s_ = 0.597, p = 0.031, Figure 3B), but not between *RBMS2* and *ERG* (r_s_ = −0.355, p = 0.234; data not shown). The non-parametric correlation between expression array and qRT-PCR results for *CRISP3* was very high (r_s_ = 0.901, p<0.001, Figure 3C), whereas the same analysis for *ERG* (r_s_ = 0.601, p = 0.029) and *RBMS2* (r_s_ = 0.641, p = 0.018) revealed a significant but smaller degree of correspondence (data not shown).

### TLDA results (validation series)

**Figure 4:**
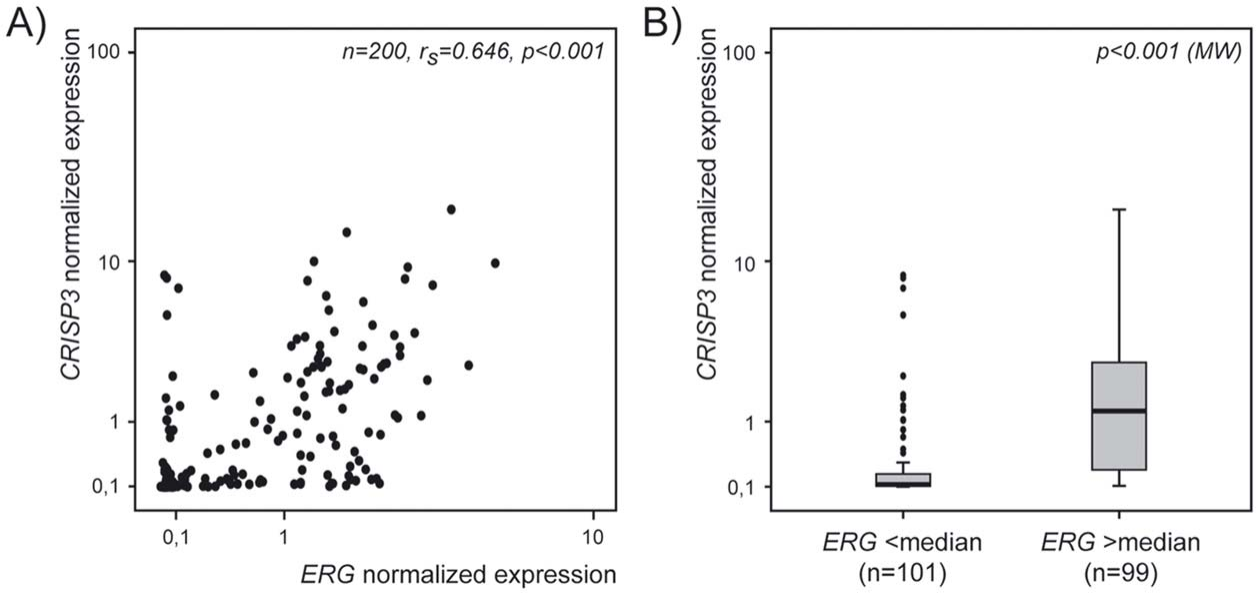
Associations between *ERG* and *CRISP3* in the validation series. A) Non-parametric correlation between expression values for *ERG* and *CRISP3*; B) Box-plots of *CRISP3* expression values grouped according to the median levels of *ERG* (Mann-Whitney U test).

Within the 200 independent carcinomas assessed using a custom-made TLDA, a positive correlation was observed between *ERG* and *CRISP3* (r_s_ = 0.646, p<0.00001, Figure 4A), but no association could be seen for *RBMS2* (data not shown). When we performed a two-group categorization of the carcinomas based on the median value of the *ERG* probe, *CRISP3* values were significantly higher in the group of samples with increased *ERG* (Figure 4B, p<0.001, Mann-Whitney U test; median 52-fold increase).

### External validation

Using the available normalized signal intensity values for both *ERG* and *CRISP3* in the 206 samples selected from Setlur *et al.* (dataset GSE8402 [18]), a significant positive correlation was found (r_s_ = 0.595, p<0.00001). When tumors were stratified according to the presence of the *TMPRSS2-ERG* rearrangement, *CRISP3* was found significantly upregulated in the fusion-positive group (Figure S3, p<0.001, Mann-Whitney U test; median 5.5-fold increase).

### CRISP3 is a direct target of ERG

**Figure 5:**
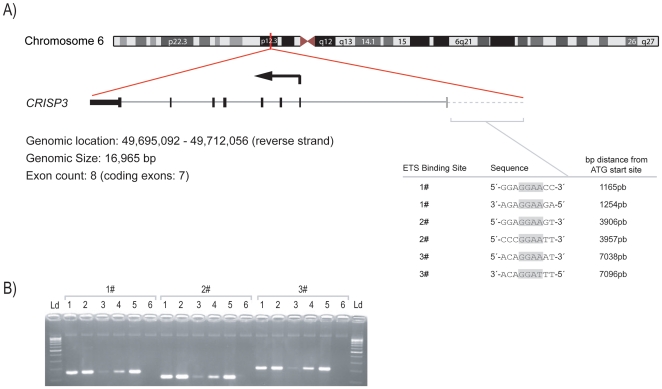
Chromatin immunoprecipitation of *CRISP3* promoter with ERG antibody. A) Schematic representation of *CRISP3* gene showing the sequence of three ETS binding sites found in the *CRISP3* promoter, each with two putative consensus binding sequences. Shadowed letters show the ETS core motif GGAA/T. B) *CRISP3* promoter amplification after chromatin immunoprecipitation with ERG antibody; specific amplification of the three ETS binding sites is shown; From left to right: Ld-100 bp DNA ladder, 1- “input” chromatin, 2- ChIP with Anti-RNA polymerase II antibody (positive control), 3- ChIP with Mouse IgG antibody (negative control), 4- ChIP with ERG antibody (#EPR3864), 5- Total DNA from VCaP cells, 6- Blank.

Using chromatin immunoprecipitation, we showed that ERG binds to the *CRISP3* promoter. From a bioinformatics approach we found 23 putative ETS binding sites in the −10 kb region of the *CRISP3* promoter (data not shown) and selected three regions for PCR analysis of the ERG-immunoprecipitated chromatin (Figure 5A). Specific amplification of the three *CRISP3* promoter regions, each containing two putative binding sequences, is shown in Figure 5B. PCR product sequences were confirmed by sequencing analysis (not shown).

### Immunohistochemistry findings

**Figure 6:**
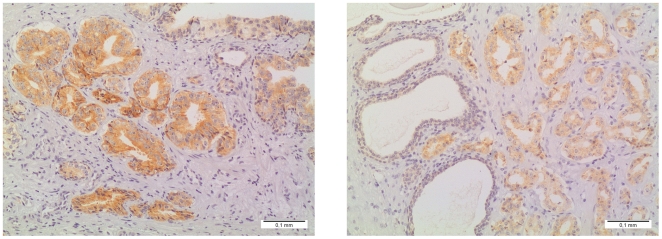
Immunoexpression of CRISP3 in prostate adenocarcinoma. The neoplastic glands demonstrate intense (*left*) to moderate (*right*) cytoplasmic staining comparatively to normal prostatic epithelium (shown in the right picture).

CRISP3 protein expression was observed in the cytoplasm of epithelial cells. A strong immunostaining was observed in the pancreatic tissue used as positive control, whereas non-malignant prostatic tissue depicted a less intense staining (not shown). In the 24 prostatectomy specimens analyzed by expression arrays, CRISP3 was classified as over-expressed in 62.5% of tumor samples (8 *ERG* positive and 7 *ERG* negative) as compared to the non-malignant prostatic tissue (Table 1, Figure 6), with the remaining showing normal/decreased protein expression. A heterogeneous staining pattern (*i.e.*, tumors containing areas with various immunostaining intensities) was found in 11 tumors (46%). No differences were observed in the staining pattern of CRISP3 between *ERG*-positive and *ERG*-negative PCa. Western-blot analysis of protein extracts obtained from two PCa samples and two prostate-derived cell lines with the CRISP3 antibody (clone LV-2A2, sc-101378) proved antibody specificity to a protein of ∼30 kDa, as expected. Interestingly, while CRISP3 was detected in both PCa samples and in the tumor-derived VCaP cells, the benign prostate cell line PNT2 showed no detectable expression (data not shown).

**Table 1:** Correlation analysis of *ERG* and *CRISP3* expression with clinico-pathological parameters in the array series (n = 24).

Clinico-pathological parameters	*ERG* rearrangement/expression			*CRISP3* expression			
	FISH		qRT-PCR*	Array	qRT-PCR*	IHC expression	
	Negative N (%)	Positive N (%)	N (Mean)	N (Mean**)	N (Mean)	Normal*** N (%)	Overexpr.N (%)
Age median (min-max)	65.5 (46-70)						
PSA at diagnosis#							
≤10	2 (22.2)	7 (77.8)	4 (170.1)	9 (20.1)	4 (65.5)	5 (55.6)	4 (44.4)
>10	4 (57.1)	3 (42.9)	3 (146.5)	7 (15.6)	3 (162.3)	2 (28.6)	5 (71.4)
	*p* = 0.152		*p* = 0.857	*p* = 0.470	*p* = 0.629	*p* = 0.280	
Gleason score							
≤7 (3+4)	6 (28.6)	15 (71.4)	9 (163.1)	21 (19.4)	9 (90.7)	9 (42.9)	12 (57.1)
≥7 (4+3)	2 (66.7)	1 (33.3)	1 (10.3)	3 (2.7)	1 (8.1)	0 (0.0)	3 (100.0)
	*p* = 0.249		*p* = 0.117	*p* = 0.206	*p* = 0.862	*p* = 0.160	
Pathological stage							
pT2	6 (60.0)	4 (40.0)	3 (3.00)	10 (9.40)	3 (3.33)	3 (30.0)	7 (70.0)
pT3	2 (14.3)	12 (85.7)	7 (6.57)	14 (14.71)	7 (6.43)	6 (42.9)	8 (57.1)
	***p*** ** = 0.019**		*p* = 0.117	*p* = 0.074	*p* = 0.183	*p* = 0.521	

*only 10 samples were available for qRT-PCR analysis.

**mean expression values (x10^3^).

***two cases that showed underexpression of CRISP3 are included.

# information is missing for 3 of the 10 cases analysed by qRT-PCR and for 8 of the 24 cases run on the Array and analysed by IHC.

Statistically significant *p*-values (<0.05) are shown in bold; no confidence intervals are indicated due to the low number of cases in the test series.

### Clinico-pathological associations

Table 1 and Table 2 show the relationship between *ERG* and *CRISP3* expression and the clinico-pathological parameters for the test and validation series, respectively. Using non-parametric tests on the qRT-PCR data from the validation series, we found a significant association between both *ERG* and *CRISP3* expression with pathological stage pT3, with *p* = 0.001 and *p* = 0.006 for *ERG* and *CRISP3*, respectively. In the array series, association of *CRISP3* expression with pT3 tumors was not statistically significant (*p* = 0.183), probably due to the low number of samples that was available for qRT-PCR analysis (n = 10). This observation is supported by the array data (n = 24) where *CRISP3* expression shows a tendency for a significant association with pT3 tumors (*p* = 0.074). Qualitative assessment of CRISP3 expression by IHC was not associated with any clinico-pathological parameter. A significant, but borderline, association was found between *ERG* expression and lower Gleason grades (*p* = 0.043), whereas *ERG* rearrangements assessed by FISH were significantly associated with pT3 staging (*p* = 0.019). The overall data, therefore, indicates that the *TMPRSS2-ERG* fusion gene and the consequent *ERG* and *CRISP3* overexpression are associated with pathological features related with locally advanced disease in patients with clinically localized prostate cancer. No significant association was found between PSA levels at diagnosis and either *ERG* or *CRISP3* expression in any of the series analyzed.

**Table 2:** Correlation analysis of *ERG* and *CRISP3* expression obtained by qRT-PCR with clinico-pathological parameters in the validation series (n = 200).

Clinico-pathological parameters	N	*ERG* expression Mean (CI)	*CRISP3 expression* Mean (CI)
Age median (min-max)	64 (49–75)		
PSA at diagnosis			
≤10	139	0.87 (0.70–1.03)	1.39 (0.93–1.85)
>10	61	0.93 (0.67–1.19)	1.28 (0.77–1.80)
		*p* = 0.527	*p* = 0.642
Gleason score			
≤7 (3+4)	156	0.94 (0.79–1.10)	1.32 (0.98–1.66)
≥7 (4+3)	44	0.68 (0.34–1.01)	1.48 (0.42–1.64)
		***p*** ** = 0.043**	*p* = 0.721
Pathological stage			
pT2	112	0.68 (0.53–0.84)	0.88 (0.60–1.16)
pT3	88	1.14 (0.89–1.39)	1.97 (1.26–2.67)
		***p*** ** = 0.001**	***p*** ** = 0.006**

Statistically significant *p*-values (<0.05) are shown in bold; CI- Confidence interval.

## Discussion

The majority of prostate carcinomas harbor recurrent fusion genes, albeit the biological mechanisms triggered by these events and their clinical significance for the patients remain mostly undetermined. Specifically, although the ETS genes involved in the rearrangements are transcription factors known to regulate key cellular processes [8], their nuclear targets in prostate tissue remain largely unknown, precluding most approaches to hinder or revert the effects of the fusion chimera. In this work we used global gene expression data from a series of prostate lesions with and without a *TMPRSS2-ERG* fusion to assess possible downstream targets of this rearrangement.

By crosschecking gene lists obtained from two-sided comparisons within different sample groups, several strong candidates emerged that could be linked to either prostate carcinogenesis in general or to overexpression of the transcription factor *ERG* in particular. The list of genes showing significant fold-changes in the presence of up-regulated *ERG* comprised mainly overexpressed candidates and included several metabolic enzymes, some of which previously found associated with *TMPRSS2*-*ERG*, such as *PLA1A* and *PLA2G7* [10], [11], [21]. A clear over-representation of membrane receptor proteins, extracellular matrix proteins and adhesion molecules was also noticeable, and in particular *MYO6*, *CHRM3* and several potassium-channel family members [11], [21]. Strikingly, the top-ranked gene on this list, *CRISP3*, showed an impressive 53-fold increase in *TMPRSS2*-*ERG*-positive cases as compared to non-malignant tissue, and an about 40-fold increase when compared to fusion-negative tumors.

The cysteine-rich secretory protein (CRISP) family is large and highly conserved among vertebrates [22]. In mammals, it comprises several members expressed predominantly in salivary glands and in the male reproductive tract, most of which under strong androgen-dependency. The rat sperm-coating protein AEG (now CRISP1), abundantly expressed in the epididymis under strict androgen control, was found implicated in the process of rat spermiogenesis, post-testicular sperm maturation, and capacitation to oocyte-sperm fusion [23]. The mouse homolog, as well as the related CRISP2 protein, were isolated and characterized shortly after from epididymal and salivary gland transcript libraries, and also found to be strongly regulated by androgens [24]. The mRNA for *CRISP3* was identified in the mouse salivary gland as an androgen dependent transcript, showing a 77% homology to *CRISP1*.

Human *CRISP3* was first described in neutrophils, but transcripts are widely distributed in exocrine glands (salivary glands, pancreas, and prostate) and also found at much lower levels in epididymis, ovary, thymus, and colon [25], [26], [27]. The human CRISP3 protein contains 245 amino acid residues and is encoded by a gene at 6p12.3, a chromosomal region that also harbors the human *CRISP1* and *CRISP2* genes. CRISP3 is an extracellular matrix protein mainly found in human plasma, saliva, seminal plasma and sweat, which can be stored intracellularly in specific compartments or granules or appear associated with membrane proteins in a glycosylated state [28], [29]. Its exact function, however, remains unclear. Based on sequence similarities to pathogenesis-related proteins in plants, cellular localization, and expression profile in neutrophils and thymus, a role as an immune response molecule has been proposed. Specifically, the presence of CRISP3 in secretory granules of neutrophils, which are rich in matrix-degradation enzymes, suggests a proteolytic role and an involvement in cellular matrix remodeling. Other seminal plasma proteases with matrix-regulation activities include TMPRSS2 (the most common fusion partner of ERG), HPN and PSA, all previously shown to be up-regulated in prostate cancer.

*CRISP3* has been previously linked to prostate carcinogenesis. Asmann *et al*. [30], using publicly available whole-genome expression data from normal and malignant prostate samples, and Ernst *et al*. [31], comparing 12,600 transcripts in 9 normal and 17 malignant prostate tissues, independently reported a significant overexpression of *CRISP3* in prostate carcinomas, being subsequently suggested as a potential prostate cancer specific biomarker [32], [33], [34]. CRISP3 expression was also tested using tissue microarrays and it was shown that patients with overexpression had a slightly higher risk of recurrence after radical prostatectomy (HR = 1.53, p = 0.010), albeit in multivariate analysis CRISP3 status did not improve the performance of existing prediction models [35]. Using a consecutive series of 200 prostatectomy samples, we found that CRISP3 overexpression at the mRNA level is associated with pathological stage pT3 (*p* = 0.006). This association was initially suggested by the array data obtained from an independent series of 24 prostatectomy samples, which also showed a significant association of CRISP3 protein overexpression with tumors with higher Gleason score (*p* = 0.009). Both associations suggest the involvement of CRISP3 in prostate cancer progression, as reported by Bjartell *et al.* [35].

Our data confirms the upregulation of *CRISP3* in prostate cancer, but further shows that *CRISP3* is under the direct control of the transcription factor *ERG*. A strong correlation between *ERG* and *CRISP3* expression was seen in both our test and validation series using different mRNA-based methodologies, and also by the external validation using the publicly available expression data from Setlur *et al.* (GSE8402) [17]. To determine if *CRISP3* was a direct target of the ERG transcription factor, we used the VCaP cell line to perform chromatin immunoprecipitation with an anti-ERG antibody, and specifically detected three putative ETS-binding-sites containing-regions of the CRISP3 promoter in the ERG-bound chromatin. To our knowledge, this is the first report showing direct regulation of CRISP3 expression by the transcription factor ERG, enhancing its relevance in the *TMPRSS2-ERG*-positive subgroup of prostate carcinomas. Interestingly, in addition to high *CRISP3* mRNA levels, also high *ERG* mRNA levels and the presence of an *ERG* fusion gene by FISH were significantly associated with pathological stage pT3, thus suggesting a role of ERG and CRISP3 in locally advanced prostate cancer in patients with clinically localized disease. However, the prognostic value of *ERG* rearrangements in prostate cancer is still controversial [15], [36], [37], [38].

Some genes showed an expression pattern suggestive of a mutually exclusive association with the *TMPRSS2*-*ERG* fusion gene. Interestingly, *SPINK1* has recently been shown to be up-regulated, in a mutually exclusive pattern, in a small percentage of *TMPRSS2*-*ERG*-negative carcinomas [39]. In the same study, the outlier profile of *ORM1* was also noteworthy and concordant with our current data [39]. Other genes were significantly over-expressed in carcinomas as compared to non-malignant tissue, but with no association to the *TMPRSS2*-*ERG* status. These genes likely play a role in prostate carcinogenesis independent of *ERG* rearrangement, and noteworthy hits based on fold-change and function are *AK5*, *RELN* and *HPN*.

Finally, a list of genes showed overexpression in *TMPRSS2*-*ERG*-negative carcinomas but an even more significant fold-increase in *TMPRSS2*-*ERG*-positive tumors, suggesting a role in malignant transformation in the prostate that is potentiated by *ERG* expression. Noteworthy hits in this subset include several previously described prostate cancer markers such as *AMACR* and *PCA3* [12]. Interestingly, most of the genes in this list are known to be under androgen-regulation, which may explain the increased levels also in malignant samples with no *ERG* fusion. *RBMS2* (nucleic acid binding protein) displayed a massive fold-change reduction in the array data in *TMPRSS2*-*ERG*-positive tumors, but this inverse correlation could not be confirmed in the larger validation series. It is thus likely that *RBMS2* reduction may play a role in malignant transformation but independently of *ERG* rearrangement.

In conclusion, we show that the *TMPRSS2-ERG* fusion gene is associated with up-regulation of several metabolic enzymes, as well as extracellular/transmembrane proteins involved in cell adhesion, matrix remodeling and signal transduction pathways. We observed a massive fold-increase of *CRISP3* in fusion-positive carcinomas as compared to non-malignant tissue or fusion-negative carcinomas and found that *ERG* genomic rearrangement and *ERG* and *CRISP3* mRNA overexpression are associated with pT3 locally advanced tumors. We further show that *CRISP3* is a direct target of overexpressed ERG, suggesting that CRISP3 may be a mediator of tumor progression driven by the *TMPRSS2-ERG* rearrangement.

## Supporting Information

Figure S1 **Genes showing different patterns of underexpression in carcinomas.** A) Genes with considerable fold-decrease in *ERG*-positive carcinomas; B) Genes with underexpression in *ERG*-negative carcinomas; C) Genes with considerable fold-decrease in carcinomas, independent of *ERG* status; D) Genes with considerable fold-decrease in ERG-negative carcinomas accompanied by an even greater underexpression in ERG-positive cancers. Abbreviations: FC(a), median fold-change between non-malignant samples (NMT) and ERG-negative carcinomas; FC(b), median fold-change between non-malignant samples and ERG-positive carcinomas; FDR, false discovery rate. The top 20 genes in each subgroup, ranked based on fold-decrease, are provided (when available).
(TIF)

Figure S2 **Box-plots representing the expression of** ***ERG*** **and** ***CRISP3*** **across sample groups.** A) Array findings (n = 30 samples); B) qRT-PCR findings (n = 13 samples). The Kruskal-Wallis (KW) non-parametric test values are indicated.
(TIF)

Figure S3 **External data.** Linearized signal-intensity values for *ERG* and *CRISP3* obtained from publicly available expression data from Setlur *et al.* for 206 prostate carcinomas: 103 with and 103 without *TMPRSS2-ERG* rearrangement (TMP-ERG^+^ and TMP-ERG^−^, respectively). The Mann-Whitney (MW) non-parametric test value is indicated.
(TIF)

Table S1 **qRT-PCR primer and probe list.**
(PDF)

Table S2 **ChIP primer list for CRISP3 promoter.**
(PDF)

Table S3 **Summarized findings in 24 prostate carcinoma samples.**
(PDF)
