# Supplementary figures and images for: Cysteine-Rich Secretory Protein-3 (CRISP3) Is Strongly Up-Regulated in Prostate Carcinomas with the TMPRSS2-ERG Fusion Gene
Source: PLoS One. 2011 Jul 21;6(7):e22317. doi: 10.1371/journal.pone.0022317 (PMC3141037; doi:10.1371/journal.pone.0022317)

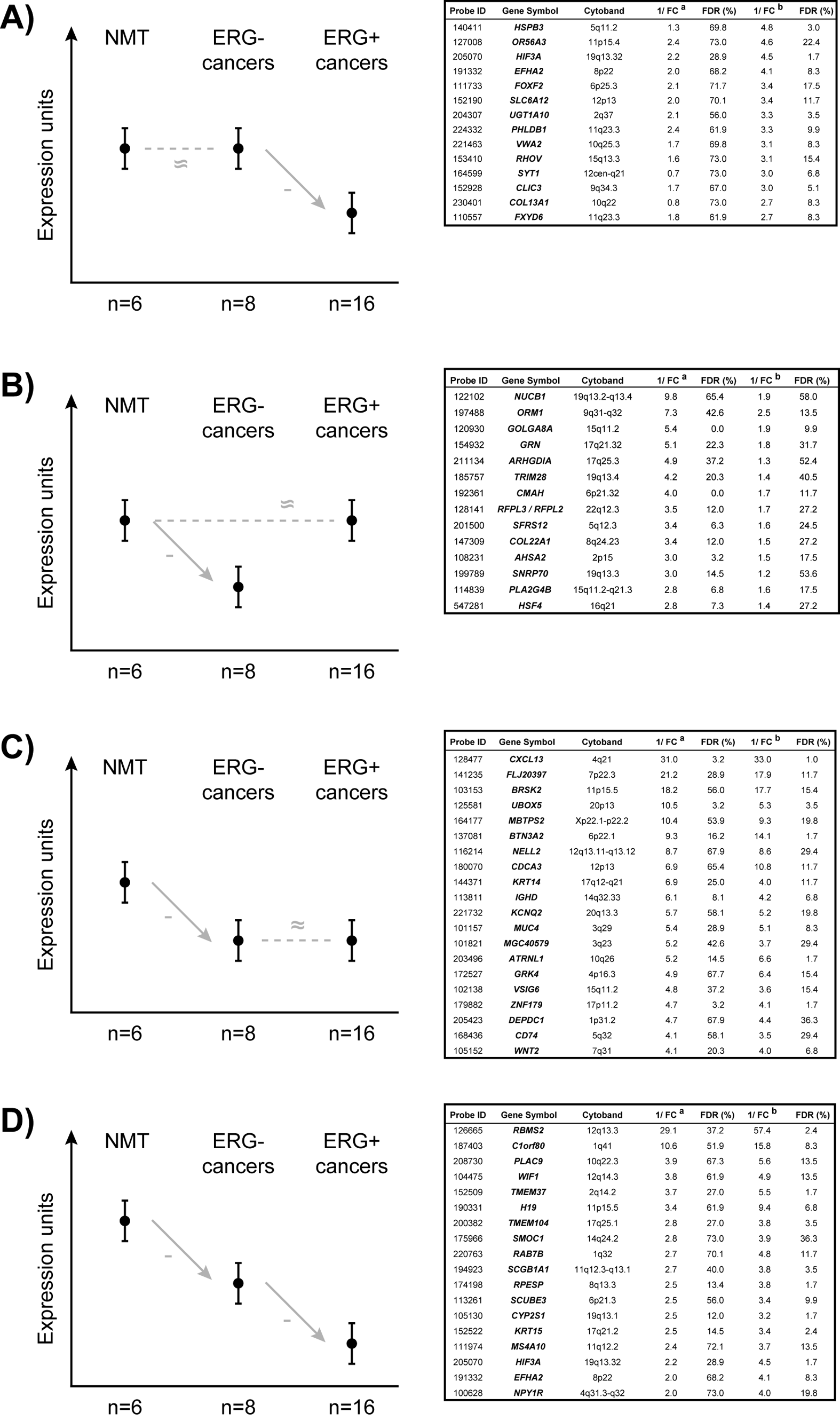

Supplement: Figure S1 — Genes showing different patterns of underexpression in carcinomas. A) Genes with considerable fold-decrease in ERG-positive carcinomas; B) Genes with underexpression in ERG-negative carcinomas; C) Genes with considerable fold-decrease in carcinomas, independent of ERG status; D) Genes with considerable fold-decrease in ERG-negative carcinomas accompanied by an even greater underexpression in ERG-positive cancers. Abbreviations: FC(a), median fold-change between non-malignant samples (NMT) and ERG-negative carcinomas; FC(b), median fold-change between non-malignant samples and ERG-positive carcinomas; FDR, false discovery rate. The top 20 genes in each subgroup, ranked based on fold-decrease, are provided (when available). (TIF) [file pone.0022317.s001.tif]

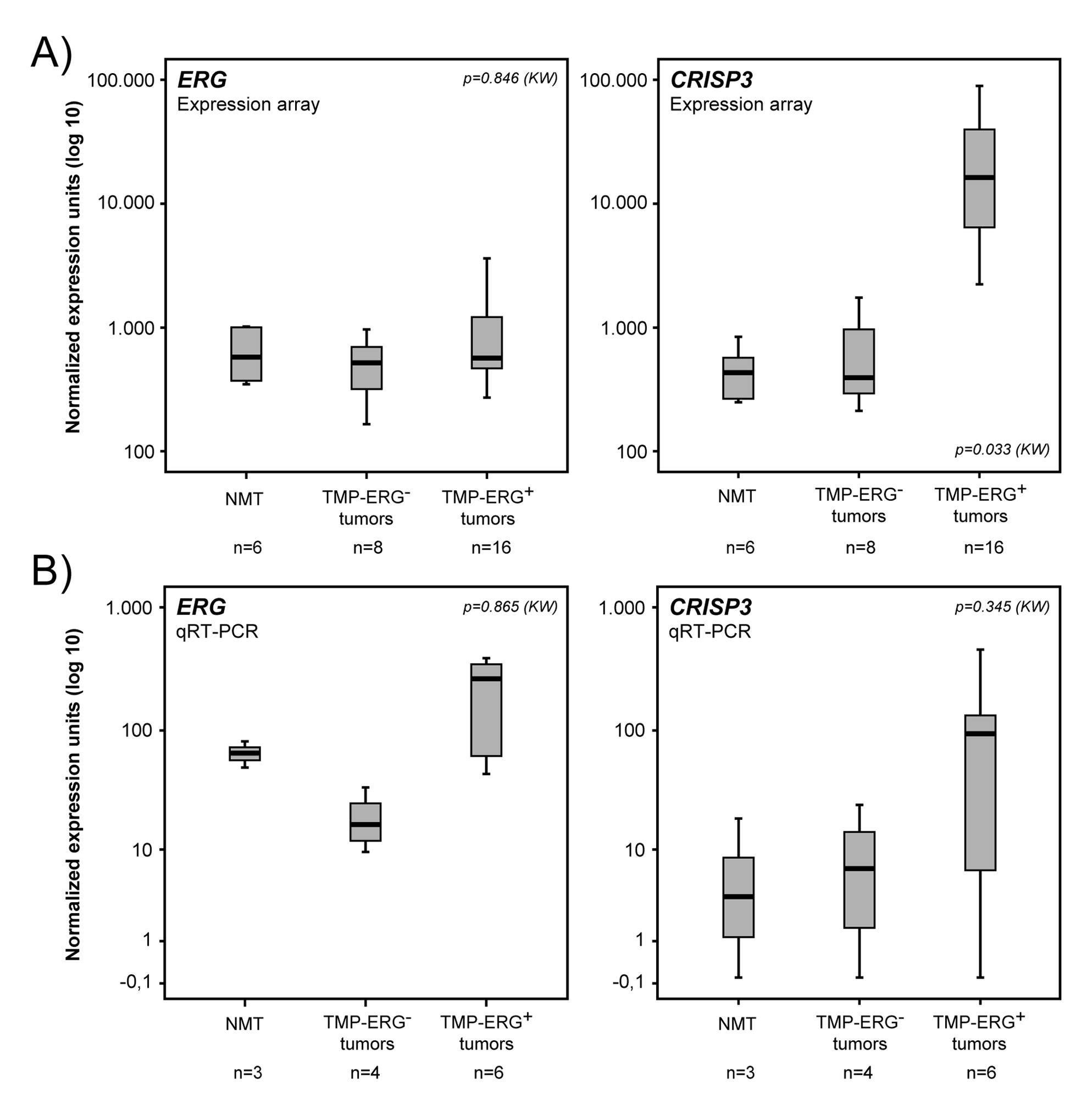

Supplement: Figure S2 — Box-plots representing the expression of ERG and CRISP3 across sample groups. A) Array findings (n = 30 samples); B) qRT-PCR findings (n = 13 samples). The Kruskal-Wallis (KW) non-parametric test values are indicated. (TIF) [file pone.0022317.s002.tif]

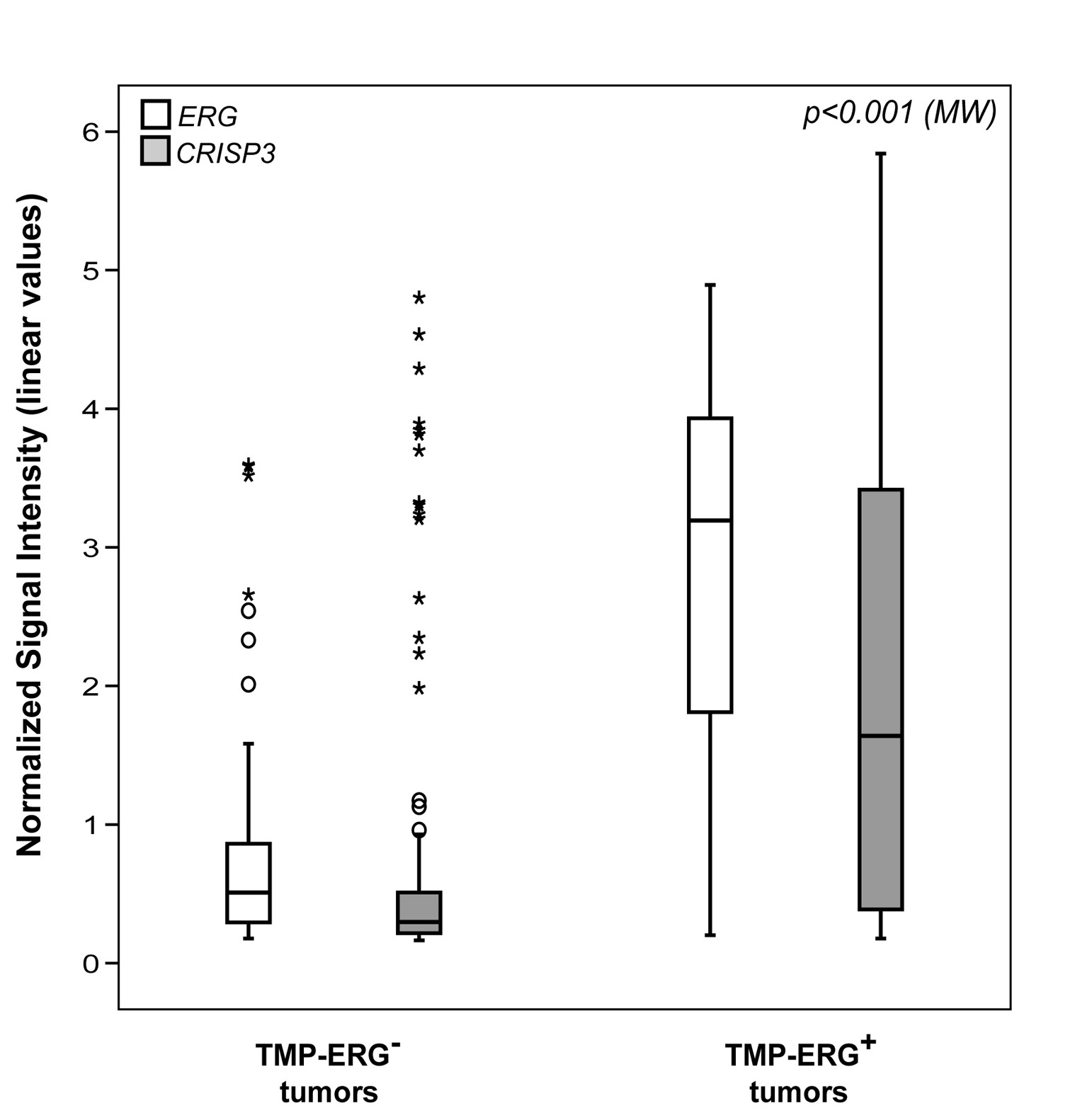

Supplement: Figure S3 — External data. Linearized signal-intensity values for ERG and CRISP3 obtained from publicly available expression data from Setlur et al. for 206 prostate carcinomas: 103 with and 103 without TMPRSS2-ERG rearrangement (TMP-ERG+ and TMP-ERG−, respectively). The Mann-Whitney (MW) non-parametric test value is indicated. (TIF) [file pone.0022317.s003.tif]
