# Supplementary material for: Cysteine-Rich Secretory Protein-3 (CRISP3) Is Strongly Up-Regulated in Prostate Carcinomas with the TMPRSS2-ERG Fusion Gene
Source: PLoS One. 2011 Jul 21;6(7):e22317. doi: 10.1371/journal.pone.0022317 (PMC3141037; doi:10.1371/journal.pone.0022317)
Supplement: Table S1 — qRT-PCR primer and probe list. (PDF) [file pone.0022317.s004.pdf]

Supp Table 3. Summarized findings in 24 prostate carcinoma samples

	FISH			Expression Arrays		Real-time PCR	
Case ID	observed pattern	ERG status	ERG group	*ERG*	*CRISP3*	*ERG*	*CRISP3*
**PCa 032**	RGB+RB+GB	positive	insertion	554.3	33947.4	nd	nd
**PCa 040**	RGB+RB	positive	deletion	3635.8	90790.3	393.2	454.8
**PCa 045**	RGB+RB+GB	positive	insertion	1082.1	6458.2	64.6	6.4
**PCa 055**	RGBx2	neg	neg	508.6	1313.6	10.3	8.1
**PCa 056**	RGBx2	neg	neg	883.5	488.2	nd	nd
**PCa 067**	GB+GB+R+R	positive*	insertion	490.0	7590.5	nd	nd
**PCa 076**	RGBx2 / RGBx4	neg	neg	522.7	1751.7	35.9	24.1
**PCa 083**	RGB+RB	positive	deletion	1446.0	7275.8	nd	nd
**PCa 087**	RGBx2	neg	neg	557.9	276.0	nd	nd
**PCa 089**	RGB+GB+RB / idemx2	positive	insertion	588.2	25552.8	nd	nd
**PCa 101**	RGB+RB+GB	positive	insertion	345.6	388.9	46.2	0.6
**PCa 114**	RGB+RB	positive	deletion	521.9	22529.4	nd	nd
**PCa 115**	RGBx2	neg	neg	202.7	310.6	19.6	5.4
**PCa 134**	RGBx2	neg	neg	973.4	316.1	16.0	0.0
**PCa 139**	RGB+RB+GB / RB+GB	positive	insertion	806.2	21581.0	296.6	74.7
**PCa 140**	RGB+RB+GB / idemx2	positive	insertion	273.8	6515.0	nd	nd
**PCa 145**	RGBx2	neg	neg	166.9	715.2	nd	nd
**PCa 148**	RGB+RB+GB	positive	insertion	454.8	47621.0	nd	nd
**PCa 151**	GRBx2	neg	neg	na	211.4	nd	nd
**PCa 155**	RGB+RB+GB	positive	insertion	1984.7	64635.8	nd	nd
**PCa 158**	RB+RB+B+B	positive	deletion	397.8	3444.9	nd	nd
**PCa 164**	RGB+RB	positive	deletion	1379.9	56585.1	244.3	117.7
**PCa 172**	RGB+RB+GB	positive	insertion	800.2	12547.5	351.9	132.3
**PCa 173**	RGB+RB+GB	positive	insertion	510.2	2246.7	nd	nd

Notes: FISH nomenclature adapted from the ISCN (2009). See probe details for expected signal patterns.
Abbreviations: nd, not determined; IHC, immunohistochemistry; (h) heterogeneous; neg, negative.
