# Supplementary material for: Cysteine-Rich Secretory Protein-3 (CRISP3) Is Strongly Up-Regulated in Prostate Carcinomas with the TMPRSS2-ERG Fusion Gene
Source: PLoS One. 2011 Jul 21;6(7):e22317. doi: 10.1371/journal.pone.0022317 (PMC3141037; doi:10.1371/journal.pone.0022317)
Supplement: Table S2 — ChIP primer list for CRISP3 promoter. (PDF) [file pone.0022317.s005.pdf]

Supp Table 1. qRT-PCR primer and probe list

Primer Name	Primer Sequence: 5'-3'	Ensembl Exon
**ERG-F**	CACGAACGAGCGCAGAGTTA	5
**ERG-R**	CTGCCGCACATGGTCTGTAC	6
**ERG-PR**	CGTGCCAGCAGATCCTACGCTATGG	5-6
**CRISP3-F**	CACAATGAACTGAGGAGAGCAGTATC	2
**CRISP3-R**	TGTCTGTAATTGCACTGGTTTGC	3
**CRISP3-PR**	CTTTGTTCCATTCCATCTTCAGCATGTTTCTG	2-3
**RBMS2-F**	AGGTTATCTCCACCCGTATCCT	5
**RBMS2-R**	TGATGGCTTCACACTTCTCTGT	6
**RBMS2-PR**	ACTCCATCCTTGCAAAGCCAACACC	5-6

Abbreviations: F, forward; R, reverse; PR, TaqMan probe, 5'*FAM*-3'*TAMRA*
