# Supplementary material for: Cysteine-Rich Secretory Protein-3 (CRISP3) Is Strongly Up-Regulated in Prostate Carcinomas with the TMPRSS2-ERG Fusion Gene
Source: PLoS One. 2011 Jul 21;6(7):e22317. doi: 10.1371/journal.pone.0022317 (PMC3141037; doi:10.1371/journal.pone.0022317)
Supplement: Table S3 — Summarized findings in 24 prostate carcinoma samples. (PDF) [file pone.0022317.s006.pdf]

Supp Table 2. ChIP primer list for *CRISP3* promoter

ETS Binding Site	Primer Name	Primer Sequence: 5'-3'
**1#**	**pCRISP3-1312-F**	CTGGTTCCTCCTGAACCTCA
**1#**	**pCRISP3-1312-R**	GCTGGTTAGGAGAAGGAAAGC
**2#**	**pCRISP3-3957-F**	CTCACGCCTTCTTACTCCCTA
**2#**	**pCRISP3-3957-R**	CCCGGAATTATATCCACTCCT
**3#**	**pCRISP3-7175-F**	TGACAGAAGGAAGGTGCAGA
**3#**	**pCRISP3-7175-R**	AGCCATTTGAGGAATCACCA

Abbreviations: F, forward; R, reverse.
